# Effects of Transforming Growth Factor Beta 1 in Cerebellar Development: Role in Synapse Formation

**DOI:** 10.3389/fncel.2016.00104

**Published:** 2016-04-27

**Authors:** Ana P. B. Araujo, Luan P. Diniz, Cristiane M. Eller, Beatriz G. de Matos, Rodrigo Martinez, Flávia C. A. Gomes

**Affiliations:** ^1^Instituto de Ciências Biomédicas, Universidade Federal do Rio de JaneiroRio de Janeiro, Brazil; ^2^Faculdade de Medicina/Departamento de Cirurgia, Universidade Federal do Rio de JaneiroRio de Janeiro, Brazil

**Keywords:** cerebellum, TGF-β1, development, excitatory synapse

## Abstract

Granule cells (GC) are the most numerous glutamatergic neurons in the cerebellar cortex and represent almost half of the neurons of the central nervous system. Despite recent advances, the mechanisms of how the glutamatergic synapses are formed in the cerebellum remain unclear. Among the TGF-β family, TGF-beta 1 (TGF-β1) has been described as a synaptogenic molecule in invertebrates and in the vertebrate peripheral nervous system. A recent paper from our group demonstrated that TGF-β1 increases the excitatory synapse formation in cortical neurons. Here, we investigated the role of TGF-β1 in glutamatergic cerebellar neurons. We showed that the expression profile of TGF-β1 and its receptor, TβRII, in the cerebellum is consistent with a role in synapse formation *in vitro* and *in vivo*. It is low in the early postnatal days (P1–P9), increases after postnatal day 12 (P12), and remains high until adulthood (P30). We also found that granule neurons express the TGF-β receptor mRNA and protein, suggesting that they may be responsive to the synaptogenic effect of TGF-β1. Treatment of granular cell cultures with TGF-β1 increased the number of glutamatergic excitatory synapses by 100%, as shown by immunocytochemistry assays for presynaptic (synaptophysin) and post-synaptic (PSD-95) proteins. This effect was dependent on TβRI activation because addition of a pharmacological inhibitor of TGF-β, SB-431542, impaired the formation of synapses between granular neurons. Together, these findings suggest that TGF-β1 has a specific key function in the cerebellum through regulation of excitatory synapse formation between granule neurons.

## Introduction

Proliferation, apoptosis, neuronal migration, and differentiation are key steps during early postnatal cerebellar development; and disturbances in any of them may lead to neurological problems such as ataxias, intellectual inability and pediatric tumors (Steinlin, 2008; Behesti and Marino, 2009; Marshall et al., 2014; Amano et al., 2016). Consequently, altered cerebellar structure and function have been associated with emotional, cognitive and social abnormalities in patients with neuropsychiatric disorders, such as autism and schizophrenia (Leiner et al., 1986; Schmahmann, 1991; Leiner, 2010).

In mice, the granule cell precursors (GCPs) migrate over the surface of the cerebellar primordia to form the EGL. After birth, GCPs proliferate extensively in the EGL, and migrate inward along the BG fibers to establish the IGL below the Purkinje cell monolayer (Altman and Bayer, 1985; Hatten, 2002).

The development of the cerebellum is controlled by intrinsic genetic programs, as well as extracellular signals such as metabolites, cytokines, interleukins, growth factors, hormones, and nutrients (Sotelo, 2004; Sillitoe and Joyner, 2007; Bosman and Konnerth, 2009), that act at specific or different steps of cerebellar development. Although, the effects of TGF-β family proteins in the CNS have been extensively studied in recent years, little is known about their expression and their effects on development and in pathological cerebellar events.

The TGF-β cytokine superfamily regulates a broad spectrum of biological responses in a large variety of cell types (Krieglstein et al., 2002; Shi and Massague, 2003). Members of this superfamily perform critical functions in the development of the CNS, including cell fate determination (Garcia-Campmany and Marti, 2007), glial differentiation (Stipursky and Gomes, 2007; Stipursky et al., 2014), embryonic and adult neurogenesis (Zhang et al., 1997; Mathieu et al., 2010; Vogel et al., 2010), and neuronal survival (Jiang et al., 2000) and migration (Siegenthaler and Miller, 2004). Recently, we demonstrated that TGF-β1 controls the formation of excitatory and inhibitory synapses in the cerebral cortex (Diniz et al., 2012, 2014b).

Some evidence supports a role for members of the TGF-β family, such as BMP, TGF-β2 and TGF-β1, in cerebellar development. BMP is important in determining GCP fate commitment during embryonic and postnatal development (Alder et al., 1999; Angley et al., 2003; Rios et al., 2004), and TGF-β2 promotes proliferation and survival of GCPs (Elvers et al., 2005). Additional evidence for a role of TGF-β1 in cerebellar development is provided by the presence of this cytokine in the cerebellum, mainly in proliferative zones during development (Sousa et al., 2004; Mecha et al., 2008). Corroborating a role for TGF-β1 in cerebellar development, TGF-β1-deficient mice showed increased neuronal death and microgliosis in the cerebellum (Brionne et al., 2003; Sousa et al., 2004; Mecha et al., 2008). Additionally, mutant mice for Smad4, Smad3 and Smad2, downstream effectors of the TGF-β1 signaling pathway, showed cerebellar development deficits, such as a decrease in the number and arborization of PCs, a reduction in cerebellar size, impaired foliation, increased apoptotic cell death, defects in granule cell (GC) maturation and migration, disturbances in the control of motor function and decreased synapse formation (Zhou et al., 2003; Wang et al., 2011).

Despite the compelling evidence for the effects of TGF-β1 on the cerebellum, the distribution of TGF-β family members and the mechanisms underlying their functions in cerebellar development are still poorly understood. Here, we characterized the expression profile of members of the TGF-β signaling pathway during cerebellar development, and we showed that TGF-β1 plays a key role in specific steps of granule neuron development: TGF-β1 does not affect GCP migration, but it induces formation of excitatory glutamatergic synapses between granule neurons.

## Materials and Methods

### Animals

Wistar rats and Swiss mice from the Federal University of Rio de Janeiro (Rio de Janeiro, Brazil) were kept under a cycle of light/dark (12 h/12 h) in controlled temperature and humidity condition with free access to water and food. All experimental protocols and handling of the animals were approved by the International Animal Care and Use Committee of the UFRJ.

### Neuronal Cultures

Neurons were prepared from cerebella derived from P7 Wistar rats and P6 Swiss mouse pups as previously described (Araujo et al., 2010). Briefly, cells were freshly dissociated from the cerebellum and plated onto glass coverslips previously coated with poly-L-lysine (MW = 389,000; 20 μg/mL; Sigma, St. Louis, MO, USA). Neuronal cerebellar cultures from rats were maintained in Neurobasal medium (Invitrogen Corporation, Carlsbad, CA, USA) with 2% B27 (Invitrogen Corporation, Carlsbad, CA, USA), penicillin, streptomycin, fungizone, and glutamine (2 mM), and cell cultures from mice were maintained in DMEM/F12 1:1 containing 10% FBS (Invitrogen Corporation, Carlsbad, CA, USA), 20 mM KCl, 36 mM glucose, penicillin, streptomycin and 10 M cytosine arabinoside (Ara-C). Cultures were maintained at 37°C in a humidified 5% CO_2_, 95% O2 air atmosphere.

### Bergmann Glia Cultures

Bergmann glia cells were prepared from cerebella derived from P5–P7 Wistar rats as previously described (Martinez et al., 2011). Cells at a density of 1,000 cells per mm^2^ were plated in culture plates, which had previously been treated with poly-L-lysine (10 μg/mL). Glial cultures were maintained for 48 h in the presence of 10 ng/mL TGF-β1 (R&D Systems, Minneapolis, MN, USA) that was added soon after plating.

### Cerebellar Explant Cultures

Cerebella explants were prepared as previously described (Santiago et al., 2001). Briefly, cerebella from P7 or E16 rats were dissected and meninges were removed. The cerebella were cut in small pieces ranging from 1 to 2 mm. The explants were plated in 96-well culture plates and coated with poly-L-lysine and laminin (40 μg/ml; Invitrogen Corporation, Carlsbad, CA, USA) for 40 min. After this period, Neurobasal medium supplemented with 2% B27 nutrient mixture (Invitrogen Corporation, Carlsbad, CA, USA) in the presence or absence of 10 ng/mL of murine TGF-β1 was added (R&D Systems, Minneapolis, MN, USA). Explants were maintained at 37°C in a humidified incubator with 5% CO_2_/95% air atmosphere for 24–48 h.

### Cerebellar Slice Cultures

Organotypic cerebellar slices were prepared as previously described (Choi et al., 2005). Briefly, cerebella were dissected from P7 Wistar rat pups and washed in Gey’s balanced salt solution. Cerebella were embedded in 1.5% agarose in PBS, and 250-μm-thick midsagittal slices were prepared using a vibrating blade microtome in an ice-cold Gey’s bath with penicillin, streptomycin and fungizone (VT1000S; Leica, Wetzlar, Germany). After removing the agarose gel, slices were transferred to 24-well culture plates with Neurobasal medium supplemented with 2% B27 nutrient mixture and maintained under agitation. Cultures were maintained at 37°C in a humidified 5% CO_2_/95% air atmosphere for 24 h.

### Reverse Transcription-Polymerase Chain Reaction

Total RNA was isolated from neuronal cultures of mouse cerebellum using TRIzol^®^ (Invitrogen, USA) according to the manufacturer’s protocol and quantified using a NanoDrop ND-1000 spectrophotometer (Thermo Fisher Scientific, USA). Two micrograms of total RNA was reverse transcribed with a High Capacity cDNA Reverse Transcription Kit according to the manufacturer’s instructions (Applied Biosystems, Foster, CA, USA). cDNA was amplified with Taq DNA polymerase in PCR buffer using a protocol from Invitrogen. Sense and antisense specific oligonucleotides for TGF-β1, TβRII, and β-actin were used. Amplification was performed with 35 cycles, and PCR products were size fractionated by electrophoresis using a 1.5% agarose gel and visualized by GelRed (Uniscience do Brasil, São Paulo, Brazil) staining as previously described (Dezonne et al., 2013; Stipursky et al., 2014).

### Quantitative Reverse Transcription-Polymerase Chain Reaction

Total RNA was extracted from mouse cerebella and neuronal cultures and reverse transcribed as described above. The primer sequences were verified using the GenBank BLAST function (Altschul et al., 1997). The primers used in this assay were: TGF-β1 (F) TAC CAT GCC AAC TTC TGT CTG GG A, (R) ATG TTG GAC AAC TGC TCC ACC TTG; TGFβ-2 (F) GGA GGT GAT TTC CAT CTA CAA C, (R) AGC GGA CGA TTC TGA AGT A; TGF-β3 (F) GAG AATT GAG CTC TTC CAG ATA C, (R) GAA AGG TGT GAC ATG GAC AG; TβRII (F) ACT GTC CAC TTG CGA CAA CCA GA A, (R) AGAAGCGGCATCTTCCAGAGTGAA; Synaptophysin (F) TGT GTT TGC CTT CCT CTA CTC, (R) TCA GTG GCC ATC TTC ACA TC; PSD-95 (F) TGC CAG ATG GAC AAG GAG ACC AAA, (R) TGT TGG CCT TGA GGTGGT AGA GTT, and β-actin (F) TGG ATC GGT TCC ATC CTG G, (R) GCA GCTCAG TAA CAG TCC GCC TAG A. Quantitative real-time RT-PCR was performed using a SYBR^®^-Green PCR Master Mix, including Ampli Taq Gold polymerase (Applied Biosystems, USA) as previously described (Dezonne et al., 2013). Reactions were performed on an ABI PRISM 7500 Real Time PCR System (Applied Biosystems). The relative expression levels of genes were calculated using the 2^-ΔΔCT^ method (Livak and Schmittgen, 2001). The amount of target genes expressed in a sample was normalized to the average of the endogenous control.

### Immunocytochemistry

The immunocytochemistry assays were carried out as previously described (Martinez et al., 2011; Diniz et al., 2012, 2014b). The primary antibodies used were rabbit anti-GFAP (Dako, Cytomation, Glostrup, Denmark; 1:1000); rabbit anti-TβRII (Millipore Corporation, Darmstadt, Germany; 1:200); mouse anti-vimentin (Promega, Madison, WI, USA; 1:300); rabbit anti-PSD95 (Cell Signaling Technology, Beverly, MA, USA; 1:100) and mouse anti-synaptophysin (Millipore Corporation, Darmstadt, Germany; 1:500). We used the following secondary antibodies: anti-IgG rabbit or mouse conjugated to Alexa Fluor 488 or 546 (Molecular Probes, Carlsbad, CA, USA; 1:1000). Nuclei were counterstained with DAPI (dilactate; Sigma, St. Louis, MO, USA) and mounted in Faramount Aqueous Mounting Medium (Dako, Cytomation, Glostrup, Denmark) on glass slides.

### Immunohistochemistry

After 24 h in culture, slices were fixed with 4% PFA and visualized with anti-BrdU as described by Choi (Choi et al., 2005). Sections were incubated for 2 h with rat anti-BrdU antibody (Roche, Mannheim, Germany; 1:150) at room temperature, followed by subsequent incubation with a secondary antibody, goat anti-rat IgG conjugated to Alexa Fluor 546 (Molecular Probes, Carlsbad, CA, USA; 1:1,000), for 40 min. Nuclei were counterstained with DAPI and mounted in Faramount Aqueous Mounting Medium on glass slides. Adult mice and 6-days-old pups were perfused with 4% PFA, and cerebella were embedded in 1.5% agarose in PBS. Then, 40-μm-thick midsagittal slices were prepared in an ice-cold PBS bath using a vibrating blade microtome (VT1000S; Leica, Wetzlar, Germany). Sections were incubated overnight with the following primary antibodies: mouse anti-calbindin (Millipore Corporation, Darmstadt, Germany; 1:200), rabbit anti-caspase-3 (Cell Signaling, Beverly, MA, USA; 1:100), mouse anti-β-tubulin III (Promega, Madison, WI, USA; 1:1,000), rabbit anti-TβRII (Millipore Corporation, Darmstadt, Germany; 1:200) and rabbit anti-caspase-3 (Cell Signaling, Beverly, MA, USA; 1:100). We used the following secondary antibodies: rabbit or mouse anti-IgG conjugated to Alexa Fluor 488 or 546 (Molecular Probes, Carlsbad, CA, USA; 1:300 and 1:500, respectively). Nuclei were counterstained with DAPI and mounted in Faramount Aqueous Mounting Medium (Dako, Cytomation, Glostrup, Denmark) on glass slides.

### Morphological and Quantitative Analyses

Morphological analyses were performed with GFAP immunostaining of BG cultures. The extensions of BG were measured between the tips of the two largest cell processes, with the assistance of the ImageJ program.

At least 10 fields were measured per well. In all cases, at least 100 randomly chosen BG cells were observed per well. The experiments were performed in triplicate, and each result represents the mean of three independent experiments as previously described (Martinez et al., 2011; Spohr et al., 2011).

### Migration Analysis of Cerebellar Explant Cultures

To analyze cell migration, explants were photographed 6, 12, 24, and 48 h after plating. At least five photographs were taken from each explant, and the distance between the explant border and the nucleus of individual migrating cells was measured with the ImageJ program. For every picture, the five most distant cells from the explant core were measured, comprising a total of 25 cells per explant. The images were taken with a Nikon TE2000 microscope.

### Migration Analysis of Cerebellar Slice Cultures

The confocal images of sagittal slices were collected with a Leica confocal microscope with a 20× objective using the following parameters: resolution was set at 1024 × 1024 pixels with 8 bit color. Multitrack settings were used with a 543 nm HeNe laser and a 488 nm argon laser. The optical slice was set at 3.0 microns. This oversampling in the z-axis improved the resolution in that axis. Four scans were made per line, with the mean of the signal recorded. The pinhole was set at 1 Airy unit. Approximately 10 images were made of each slice, and three slices were used for each experimental condition. Slices were transferred to 24-well culture plates with Neurobasal medium supplemented with 2% B27 nutrient mixture and maintained under agitation. 5-bromo-2′-deoxyuridine (BrdU; 16 M, Sigma, St. Louis, MO, USA) was added to the medium for 4 h, and slices were washed twice with fresh medium and cultured in Neurobasal medium supplemented with 2% B27 nutrient mixture in the presence or absence of 10 ng/mL of murine TGF-β1 (R&D Systems, Minneapolis, MN, USA) or 10 ng/mL of murine EGF (Invitrogen Corporation). Cultures were maintained at 37°C in a humidified 5% CO_2_/95% air atmosphere for 24 h. BrdU-positive cells in the untreated (control) slices were confined to the EGL. This is consistent with the understanding that BrdU is incorporated into cells passing through the S phase and that cycling neural cells are distributed in the EGL (Sotelo, 2004). Two hours after addition of BrdU, BrdU-labeled cells migrated from the EGL to form the IGL. The BrdU-positive cells that left the EGL were counted with the ImageJ program and normalized to the total number of labeled cells.

### Puncta Analysis

The neuronal cells were maintained for 5 days followed by 24 h in the presence of DMEM-F12 medium supplemented with 10 ng/mL of TGF-β1 (R&D Systems, Minneapolis, MN, USA) or with 10 M of a pharmacological inhibitor of TβRII, SB-431542 (Sigma Chemical Co., St. Louis, MO, USA). After fixation with 4% PFA for 15 min, the cultures were permeabilized with 0.2% Triton X-100 for 5 min at room temperature, and non-specific sites were blocked with 10% bovine serum albumin (Sigma, St. Louis, MO, USA) for 1 h before immunoreactions with the antibodies against the pre- and post-synaptic proteins, synaptophysin and PSD-95, respectively. Neurons were identified using the DAPI stain. After capturing 10–15 images per experimental condition, the green and red channels were aligned and quantified using the Puncta Analyzer plug-in from NIH ImageJ as previously described (Christopherson et al., 2005; Diniz et al., 2012). Experiments were performed in duplicate, and each result represented the mean of at least three independent neuronal cultures.

### Immunoblotting Assays

Protein concentration in cell extracts was measured with the BCATM Protein Assay Kit (Cole-Parmer Canada, Inc., Montreal, QC, Canada). Forty micrograms of protein per lane was electrophoretically separated in 12% sodium dodecyl sulfate polyacrylamide gels (SDS-PAGE). After separation, the proteins were electrotransferred to a Hybond-P polyvinylidene difluoride transfer membrane (Amersham Biosciences, Little Chalfont, Buckinghamshire, UK) for 1.5 h. Non-specific sites were blocked by membrane incubation in Tris-buffered saline-Tween 20 (TBS-T; Merck, Darmstadt, Germany) containing 5% milk for 1 h. Membranes were incubated in blocking solution containing primary antibodies overnight, followed by a 2 h incubation with peroxidase-conjugated secondary antibodies. Proteins were observed using the enhanced chemiluminescence detection system (Super Signal West Pico Chemiluminescent Substrate; Pierce Biotechnology, Milwaukee, WI, USA), and PVDF membranes were exposed to autoradiographic films. Primary antibodies were mouse anti-synaptophysin (anti-mouse, Millipore Corporation, Darmstadt, Germany; 1:500), rabbit anti-PSD-95 (Cell Signaling Technology, Beverly, MA, USA; 1:1,000), mouse anti-Smad2/3 (Santa Cruz Biotechnology, Inc.; 1:1000), rabbit anti-P-Smad2/3 (Millipore Corporation, Darmstadt, Germany; 1:1000) and rabbit anti-cyclophilin (Sigma-Aldrich, St. Louis, MO, USA; 1:1000). Secondary peroxidase-conjugated antibodies were goat anti-rabbit IgG and goat anti-mouse IgG (Amersham Biosciences, Little Chalfont, Buckinghamshire, UK; 1:3,000) as previously described (Diniz et al., 2012).

### Statistical Analysis

Student’s *t*-test was used when only two conditions were compared or one-way analysis of variance (ANOVA) followed by a non-directional Tukey’s post-test when statistical significance was achieved using the GraphPad Prism software version 5.0 (GraphPad Software, La Jolla, CA, USA). A confidence interval of 95% was used, and a *P*-value of <0.05 was considered statistically significant. Densitometry of blotted gels was assessed using the program Un-Scan-It gel version 6.1 (Silk Scientific, Inc., Orem, UT, USA). Data are reported as the mean ± SEM, and error bars in the graphs represent SEM. All experiments were performed at least in duplicate as previously described (Martinez et al., 2011).

## Results

### Expression of TGF-β and TβRII During Early Postnatal Cerebellar Development: Granule Cells Activate the TGF-β1 Signaling Pathway

**FIGURE 1:**
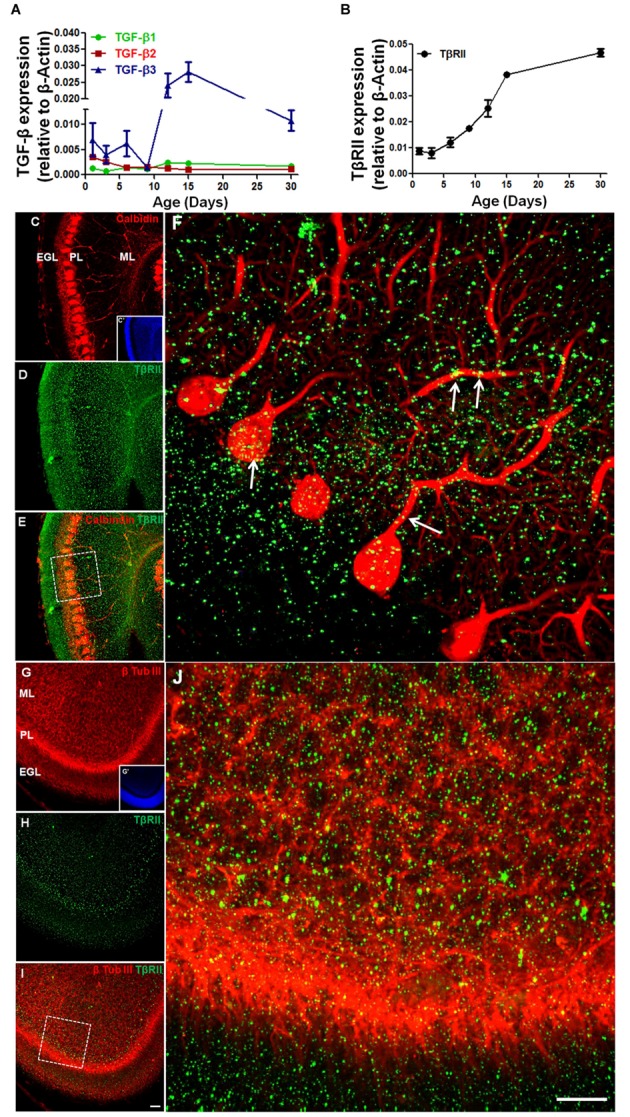
**The expression of TGF-β and TGF-β receptor during early postnatal cerebellar development.** mRNA levels of TGF-β1, TGF-β2, TGF-β3, **(A)** and TβRII **(B)** were analyzed by qPCR in the cerebellum of mice at different postnatal ages (1, 3, 6, 9, 12, 15, and 30 days). Data are presented as the mean ± error relative to β-actin gene expression (*n* = 3). TβRII distribution was analyzed in sagittal sections of the postnatal day 6 (P6) mouse cerebellum stained with anti-calbindin **(C)**, anti-β-tubulin III **(G)** and anti-TβRII **(D,H)**. Note that TβRII colocalizes with calbindin **(E,F)** in the PCL and with the β-tubulin III neuron marker in the ML **(I,J).** Scale bar: 40 μm **(C,G)** and 20 μm **(F,J)**.

Although, the expression of TGF-β and its receptor has been characterized in the developing cerebral cortex (Stipursky et al., 2014), the cerebellar expression profile of members of the TGF-β family is unknown. Using real-time PCR, we evaluated the expression of TGF-β1, TGF-β2, TGF-β3, and TGF-β receptor (TβRII) in the mice cerebellum during the development. Expression of TGF-β1 was low in the early postnatal days (P1–P9) and increased after P12, remaining constant until adulthood (P30). In contrast, the expression of TGF-β2 was higher in the first postnatal days (P1–P3) and decreased until P6–P9, remaining constant until adulthood. The expression of TGF-β3 was the highest of the three members of the TGF-β family. It strongly increased from the P9 to the P15, followed by a small decrease in adulthood (**Figure 1A**). TβRII expression increased from P3 to P30 (**Figure 1B**).

To determine if Purkinje cells and GC precursors express TβRII, we studied the TβRII distribution in the cerebellum of P6 mice. Immunocytochemistry assays identified TβRII in both cell types *in vivo* (**Figures 1C–J**).

Members of the TGF-β family bind to a heteromeric receptor complex consisting of TGF-β receptor (TβR) types I and II, which thus leads to phosphorylation and activation of the Smad proteins, downstream cellular effectors of TGF-β signaling.

**FIGURE 2:**
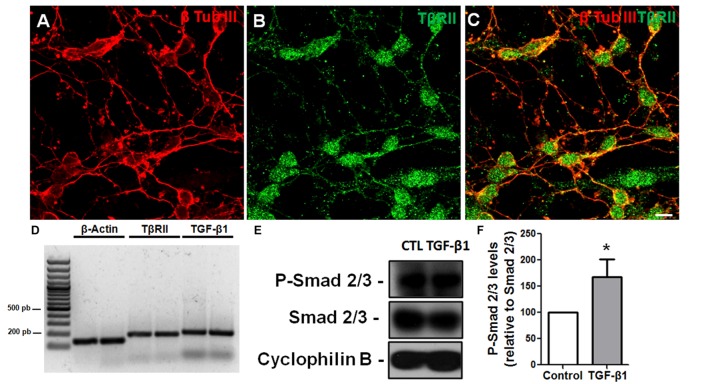
**Granule cells are responsive to TGF-β1 *in vitro.*** Granule cells obtained from P6 mice were cultured for 24 h, and the distribution of TβRII and levels of TβRII and TGF-β1 were evaluated by immunocytochemistry **(A–C)** and PCR **(D).** Confocal microscopy identified the presence of TβRII throughout the neuronal membrane **(A–C).** Scale bar: 20 μm. PCR assays demonstrated that GCs express TβRII and TGF-β1 *in vitro* **(D)**. Treatment of GCs with TGF-β1 (10 ng/mL) for 15 min increased the levels of *p*-SMAD, a hallmark of TGF-β1 pathway activation **(E,F)**. ^∗^*P* < 0.05.

To assess the response of GCs to TGF-β, we used GC-enriched cultures derived from cerebella of P6 mice and analyzed TβRII distribution and Smad phosphorylation. Using immunocytochemistry, we found that GCs express the TβRII, with the typical punctuated distribution expected for this protein (**Figures 2A–C**). This result was confirmed by PCR assays that showed that GCs express both the receptor for TGF-β1 and the factor itself (**Figure 2D**). Consistent with the idea that GCs are targets of TGF-β1, treatment of GCs with 10 ng/mL of TGF-β1 increased the levels of *p*-Smad, suggesting that these cells are responsive to TGF-β1 (**Figures 2E,F**). Together, these data suggest that GCs are either a source or target of TGF-β1.

### TGF-β1 Does Not Affect Granule Cell Migration

**FIGURE 3:**
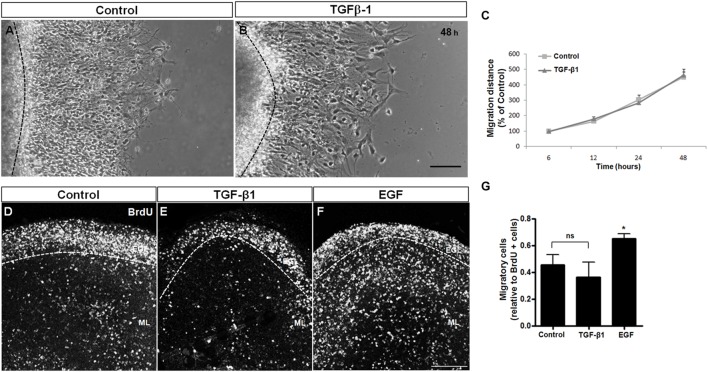
**TGF-β1 does not induce neuronal migration.** Cerebellar neuronal migration was assessed with 2 approaches: cerebellar explants **(A–C)** and cerebellar slices **(D–G). (A–C)** Cerebellar explants derived from P7 rats were cultured for 48 h in Neurobasal medium supplemented with 2% B27 alone **(A)** or in the presence of 10 ng/mL of TGF-β1 **(B)**. After that, neuronal migration distance was measured at 6, 12, 24, and 48 h **(C). (D–G)** Cerebellar slices derived from P7 rats were cultured in Neurobasal medium supplemented with 2% B27 alone **(D)** and in the presence of 10 ng/mL of TGF-β1 **(E)** or 10 ng/mL of EGF **(F)**. Neuronal migration was evaluated after 24 h. The results represent the mean of 3 independent experiments. TGF-β1 did not interfere with neuronal migration in either methodological approach. Scale bars: 100 μm. ^∗^*P* < 0.05.

TGF-β1 has been associated with neuronal migration in the cerebral cortex (Siegenthaler and Miller, 2004). To evaluate the effect of TGF-β1 in cerebellar neuronal migration, we used two approaches: cerebellar explant culture assays (**Figures 3A–C**) and organotypic cultures (**Figures 3D–G**), both well-known models used to study neuronal migration (Mancini et al., 2009; Martinez et al., 2011; Kapfhammer and Gugger, 2012; Uesaka et al., 2012; Pritchard et al., 2014).

Cerebellar explants were treated with TGF-β1 for 48 h, and the migration was measured during this time period. As shown in **Figure 3C**, TGF-β1 was unable to modify the migration distance during the time course, suggesting that TGF-β1 does not modulate the migratory process in the cerebellar cortex. To confirm these results, cerebellar slices were treated with TGF-β1 (10 ng/ml) or EGF (10 ng/ml, positive control; Caric et al., 2001; Carrasco et al., 2003; Martinez et al., 2011) for 24 h, and the distribution of BrdU-positive cells that left the EGL (migrating neurons) was analyzed. As shown in **Figures 3D–G**, TGF-β1 treatment did not affect the number of BrdU-migrating cells in cerebellar slices, whereas EGF, which was previously reported to induce cerebellar neuronal migration (Martinez et al., 2011), increased the number of migratory cells by 48.8% (**Figure 3G**). These findings further support the hypotheses that TGF-β1 does not modulate GC migration.

In the cerebral cortex, TGF-β1 has been reported to induce neuronal migration in a concentration-dependent manner. Therefore, we tested three different concentrations of TGF-β1 (2.5, 10, and 40 ng/mL) and analyzed the explant migration 48 h after plating. TGF-β1 did not enhance cerebellar neuronal migration at any of the concentrations (Supplementary Figure S1).

During development, GCPs migrate along BG radial fibers to reach the IGL. Alterations in BG morphology severely impair neuronal migration (Rakic and Sidman, 1973; Hatten et al., 1986). We thus analyzed BG glial morphology in the presence of TGF-β1 in cell explants and BG cell cultures. In these models, BG cells acquired a radial morphology, extending their fibers out of the core of the explants (Martinez et al., 2011). Measurement of the length of GFAP processes revealed that TGF-β1 did not affect BG glial extension (Supplementary Figure S2).

Together, these findings indicate that TGF-β1 does not induce GC migration and BG radialization in the cerebellum.

### TGF-β1 Induces Synapse Formation between Cerebellar Granule Cells

**FIGURE 4:**
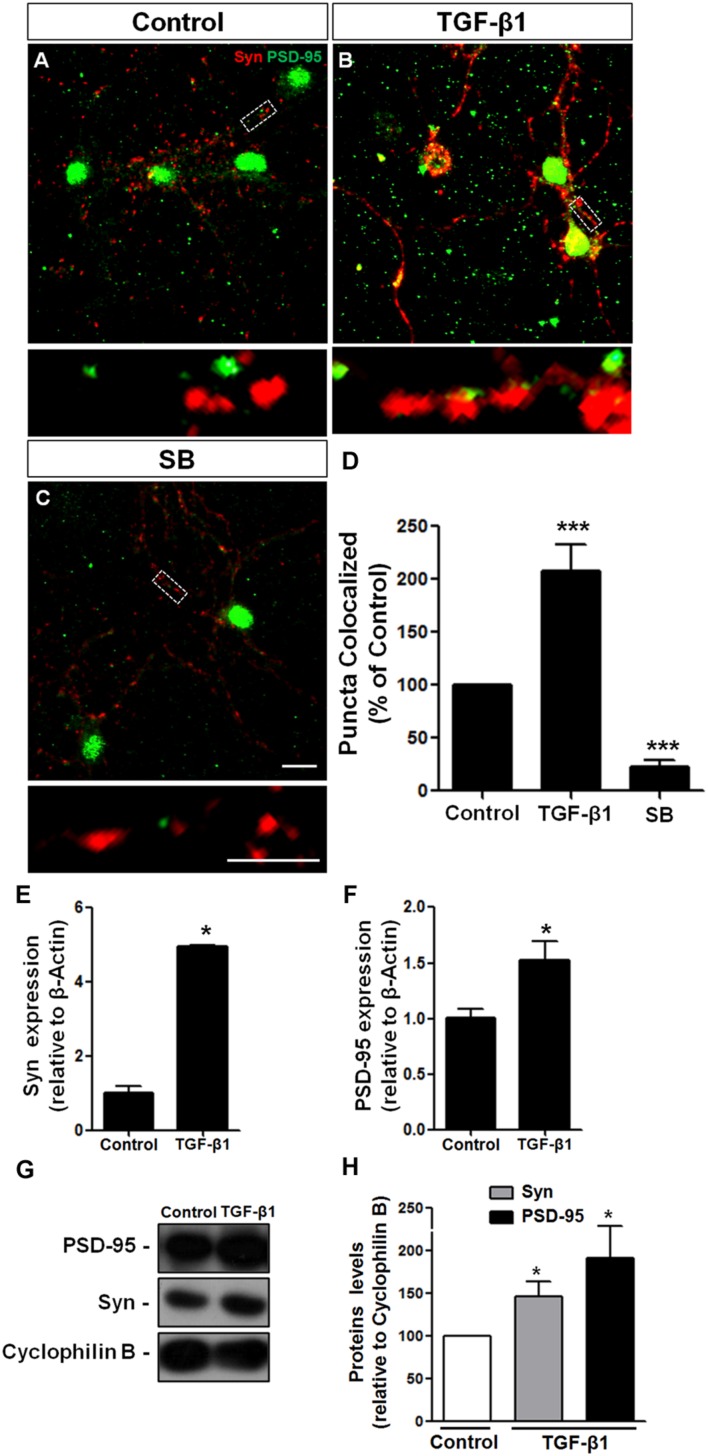
**TGF-β1 induces synapse formation between cerebellar neurons *in vitro.*** GCs derived from mice cerebella of P6 were cultured for 5 days in the presence of DMEM-F12 medium **(A)** and supplemented with 10 ng/mL of TGF-β1 **(B)** or the pharmacological inhibitor of TβRII, SB-431542, **(C)** for an additional 24 h. Synapse formation was evaluated by counting the number of synaptophysin/PSD-95 colocalization puncta **(D).** Levels of synaptic proteins were evaluated by qPCR assays **(E,F)** and Western blotting **(G,H).** Scale bars, 10 μm. ^∗^*P* < 0.05. TGF-β1 increased the number of excitatory cerebellar synapses and the levels of the pre- and post-synapse proteins, synaptophysin and PSD-95, respectively. ^∗∗∗^*P* < 0.001.

TGF-β1 has been strongly implicated in synapse formation in invertebrates (Chin et al., 2002) and the vertebrate nervous system (Krieglstein et al., 2012) We recently demonstrated that TGF-β1 increases the excitatory and inhibitory synapse formation in cerebral cortical neurons (Diniz et al., 2012, 2014b). To investigate the possibility that TGF-β1 acts as a synaptogenic molecule for glutamatergic cerebellar neurons, we cultured mouse neurons in the presence of TGF-β1 and SB-431542, a pharmacological inhibitor of the TGF pathway, and analyzed synaptic molecules by immunocytochemistry, Western blotting and qRT-PCR assays. Synapse formation was firstly evaluated by quantification of double-immunolabeled puncta for the pre- and post-synaptic proteins, synaptophysin and PSD-95, respectively. Treatment of granule neurons with TGF-β1 doubled the number of synapses as revealed by quantification of synaptophysin/PSD95 double-immunolabeled puncta (**Figures 4A–D**). The addition of SB-431542 decreased the formation of synapses between granular neurons below the levels of the control, suggesting that endogenous TGF-β is important to maintain synapses in granular cell cultures (**Figures 4C,D**). Levels of pre- and post-synaptic proteins were also increased by TGF-β1 as shown by qPCR (**Figures 4E,F**) and Western blotting (**Figures 4G,H**).

Together, these data demonstrate that TGF-β1 induces formation of morphologically organized synapse in GCs *in vitro*.

## Discussion

Here, we characterized the expression profile of the members of the TGF-β family and its receptor during cerebellar development. We found that the granule neurons are potentially responsive to TGF-β1 because they express the machinery for TGF-β1 signaling. We found that TGF-β1 regulates a specific developmental stage of granular cell development, the formation of excitatory synapses.

In the CNS the expression of TGF-β isoforms and their receptors has been well-characterized (Bottner et al., 1996; De Groot et al., 1999; Vincze et al., 2010), but the distribution and role of different isoforms in cerebellar development are still unclear. Developmental defects occur when different isoforms of TGF-βs are knocked out (Dunker and Krieglstein, 2002). Recently, it has been demonstrated that Smad2 protein disruption leads to aberrant cerebellar development (Wang et al., 2011), indicating that TGF-β may play a key role in cerebellar development. During early metencephalon-myelencephalon development (E13.5), TGF-β1 is expressed in the EGL and the cerebellar nuclei primordium, suggesting that it may be involved in the initiation of cerebellar primordium differentiation (Mecha et al., 2008). Between the second and fourth postnatal days (P2–P4) in the mouse cerebellum, GCPs proliferate (Wechsler-Reya and Scott, 1999), reaching a peak from the fifth to eighth (P5–P8) postnatal day (Hatten, 1985). Between birth and the end of the second postnatal week, GCPs stop proliferating and migrate to the interior regions of the EGL, where they extend parallel fibers and migrate along the radial fibers of BG cells (Edmondson and Hatten, 1987) through the PCs to form the IGL. In this period, we observed a higher expression of TGF-β3, and we did not observe differences in the expression of TGF-β1 and 2 (**Figures 1A,B**). This finding suggests that the TGF-β3 isoform is important in this event.

In the adult cerebellum, TGF-β2 has been reported to have the highest expression of the three TGF-β isoforms, especially in the PC layer; only a few scattered cells express TGF-β1 in all layers, and TGF-β3 is absent (Vincze et al., 2010). Contrary to these findings, we observed an increase in TGF-β3 expression compared with the other isoforms in the adult mouse cerebellum. This discrepancy is likely due to the sensitivity of the techniques used. The qPCR technique is sensitive enough to allow quantification of low levels of mRNA from a single cell (Heid et al., 1996; Tsuzuki et al., 2001) compared with *in situ* hybridization, where the detection levels are in the range of 10–20 copies of mRNA per cell (Nakamura, 1990).

TGF-βs bind to their receptors TβRI and TβRII, leading to phosphorylation of Smad2-3, followed by complex formation with Smad4 and nuclear translocation, thus modulating TGF-β target gene transcription (Massague, 1992, 1998). In the present study, we found that cerebellar development is followed by increased expression in TβRII (Sotelo, 2004; Sillitoe and Joyner, 2007; Marzban et al., 2014). In addition, we identified this receptor in Purkinje neurons and GCPs (*in vitro* and *in vivo*), indicating that these cells are potentially responsive to TGF-β.

At the end of embryonic stage, the EGL is formed, and GCPs proliferate. Radial migration of these cells starts later, at 5–7 days after birth (Sotelo, 2004). BG cells—radial glia cells in the cerebral cortex—act as scaffolds for neuronal migration in the cerebellar cortex. Many molecules have been shown to be involved in BG differentiation and migration in the cerebellum, such as astrotactin (Adams et al., 2002), neuregulin (Rio et al., 1997), gangliosides (Santiago et al., 2004), Notch (Weller et al., 2006), FGF9 (Lin et al., 2009), β1-integrin (Blaess et al., 2004; Belvindrah et al., 2006) and EGF (Martinez et al., 2011). Here, we investigated whether TGF-β1 is another molecule that influences cerebellar migration and BG morphology.

In the CNS, TGF-β family members are expressed by cells directly involved in migratory processes, such as neurons, radial glia (Siegenthaler and Miller, 2004), oligodendrocytes, microglia (Unsicker and Strelau, 2000), and proliferative precursors (Mecha et al., 2008). Recently, expression of TGF-β1 was found in all neuron-populated areas, such as the cortical layers and the deep nuclei of the cerebellum (Vincze et al., 2010).

The role of TGF-β1 in neuronal migration in the cerebellum has been a subject of controversy. Loss of TGF-β1 leads to increase of cell death in mouse brains (Brionne et al., 2003). Decrease of TGF-β1 and Smad 4 mRNA expression by an agonist of LXR (Liver X receptor, members of the nuclear receptor superfamily of ligand-activated transcription) leads to delay in differentiation of BG cells and increase in migration of granule neurons (Xing et al., 2010); thus suggesting an inverse correlation between TGF-β1 and neuronal migration in the cerebellum. On the other hand, the knockout of Smad2 in CNS revealed increased apoptotic cell death, defects in GC maturation, migration, abnormal PC dendritogenesis, and decreased synapse formation in the cerebellum (Wang et al., 2011). The loss of Smad4 resulted in reduction in cerebellar size, defects in cerebellar cytoarchitecture and hyperactivity (Zhou et al., 2003; Fernandes et al., 2012). Despite evidence suggesting a role for TGF-β in cellular migration, exogenous addition of TGF-β1 has not altered neuronal migration in our study. Together with ours, these results suggest that TGF-β1 might distinctly affect neuronal migration depending on stage of development, CNS structure, concentration, and model. The mechanism by which TGF-β1 impacts neuronal migration in the cerebellum is yet to be fully determined.

There are many possible explanations for the lack of effects described here; (1) first, the heterogeneity between regions might be responsible (cerebellar cortex vs. cerebral cortex), and (2) second, although the explant model mostly preserves the cortical architecture, including glial-assisted migration, it is possible that other important factors influencing migration, such as the position of the meninges, the interaction with other cell types and the concentration gradient of some factors, are lost.

Although, cerebellar development is regarded as mainly postnatal, some important cellular events, such as the tangential migration of the granular precursors from the rhombic lip and the beginning of the extensive proliferation of the granular precursors at the EGL, do occur at an earlier period, during the embryonic stages. TGF-β1 is expressed at the beginning of EGL formation (Mecha et al., 2008). To study whether TGF-β1 could influence cerebellar migration at an earlier development period, embryonic explants (E16–E18) were treated with TGF-β1. Even under these circumstances, TGF-β1 did not interfere with cerebellar migration. Studies using TGF-β1- or Smad 4-deficient mice clearly demonstrated a key role for this factor in maintaining embryonic neuronal integrity and survival. This is particularly true for mutations present in this gene in the early stages, in which very early (E 7.5) lethality is found (Sirard et al., 1998). Later mutations in Smad 4 (Zhou et al., 2003) or mutations in other specific genes, such as TGF-β1, result in much less prominent alterations, such as hyper-myelination in the presence of a normal overall structure of the CNS (Kulkarni et al., 1993; Day et al., 2003).

Radial migration in the cerebellum and cerebral cortex is directly dependent on the radial morphology of BG and radial glia cells, respectively. Several factors have been shown to promote and maintain the radial phenotype of these cells, such as Notch signaling, neuregulins, adhesion proteins, and extracellular matrix molecules (Adams et al., 2002; Schmid et al., 2003; Belvindrah et al., 2006; Patten et al., 2006; Zheng and Feng, 2006; Birchmeier, 2009). EGF has recently been shown to have remarkable effects on BG elongation and cerebellar neuronal migration (Martinez et al., 2011). Here, we showed that TGF-β1 did not promote changes in BG morphology, despite the expression of TβRII by these cells (data not shown). These results are in accordance with our findings related to neuronal migration.

The lack of effects in neuronal migration and BG elongation does not rule out an important role for this molecule in CNS development. Several studies report key roles for TGF-β1 during the steps of nervous system development, such as a glial differentiation, neuronal migration, radial glia commitment, blood–brain barrier formation, and neurogenesis (De Sampaio et al., 2002; Schmid et al., 2003; Battista et al., 2006; Stipursky and Gomes, 2007; Romao et al., 2008; Vogel et al., 2010). Our data, however, highlight the specificity and pleiotropic actions exhibited by TGF-β family members. We previously showed that astrocytes derived from different brain regions, e.g., cerebral cortex, midbrain and cerebellum, distinctly respond to TGF-β1: whereas the GFAP gene from the cerebral cortex and midbrain astrocytes is activated by TGF-β1, the GFAP gene from the cerebellum is not (Sousa et al., 2004; Romao et al., 2008). Together, these data indicate a possible role for TGF-β1 in the nervous system and highlight the heterogeneity of the cues that govern cerebella and cerebral cortex development.

Granular cells are the major excitatory cell type found in the cerebellum (Voogd et al., 1996). They project their axons in the ML, where they bifurcate as parallel fibers perpendicular to the plane and make synaptic contact with several dendrites of Purkinje cells. The parallel fibers of excitatory GCs also make contact with the dendrites of the Golgi, stellate, and basket cells (Sillitoe and Joyner, 2007). Synaptogenesis is a process that involves neurite extension, contact site recognition, synapse specialization, synapse maturation and maintenance (Garner et al., 2006). Our knowledge of the genetic regulation of cerebellar synaptogenesis is still very limited.

Members of the TGF-β family have been implicated in the formation and function of excitatory synapses in vertebrates and invertebrates (Fukushima et al., 2007; Feng and Ko, 2008; Diniz et al., 2012) TGF-β1 effects on synapses are cell context dependent. While TGF-β1 is not able to induce formation of excitatory synapses between retinal ganglion neurons (Christopherson et al., 2005), it induces synapses between hippocampal neurons (Bae et al., 2011), cerebral cortex neurons (Diniz et al., 2012) and at the neuromuscular junction (Feng and Ko, 2008). Here, we found that addition of TGF-β1 induces the formation of excitatory synapse between granular neurons. In agreement with this result, loss of function of endogenous TGF-β in culture (by pharmacological blockade) decreased endogenous synapse number. While *in vitro* experiments showed that both neurons (de Luca et al., 1996) and astrocytes (Sousa et al., 2004) synthesize TGF-β1, the exact source of TGF-β1 in the cerebellum still requires further study.

TGF-β1 over-expression in astrocytic cells *in vivo* leads to intense hippocampal astrogliosis and an increase in the levels of AMPA and NMDA receptors subunits; however, TGF-β-deficient mice present intense astrogliosis, dendritic spine density loss, dysfunction in LTP and LTD, and deficiency in astroglial glutamate transport proteins (Koeglsperger et al., 2013). Our group has demonstrated that astrocytic TGF-β1 is able to induce excitatory and inhibitory synapse formation in cortical neurons through distinct signaling pathways, thus suggesting a role for this factor in the balance between excitatory and inhibitory inputs (Diniz et al., 2012, 2014a,b). Corroborating our findings, Smad2 knockout mice exhibited behavioral abnormalities in motor coordination, abnormal Purkinje cells dendritogenesis and decreased inhibitory synapse formation in the cerebellum, indicating the role of TGF-β signaling in inhibitory (Wang et al., 2011) and excitatory synaptic formation in the cerebellum (described in the present study).

Granular neurons play a pivotal role in the control of information flow between cerebellar inputs and outputs through synaptic interactions with mossy fibers and Purkinje neurons, respectively (Medina et al., 2000; D’Angelo and De Zeeuw, 2009; D’Angelo, 2014). Between P3–P7, when mossy fibers form globular en passant boutons in the IGL and send fine branches upward into the PCL (Mason and Gregory, 1984), we observed that the expression of TGF-β1 increases concomitantly with the receptor expression (TβRII). Our results show that TGF-β1 expression peaked at P12, the period in which mossy fibers form large terminals among small sets of granule neurons, the synaptic glomeruli (Hatten and Heintz, 1995). During the third postnatal week, when the GCs undergo synaptogenesis (Altman, 1972), we observed high expression of TβRII, suggesting that TGF-β1 signaling may be involved in this event.

Several mechanisms have been proposed to be implicated in synaptic formation, such as regulation of neurite outgrowth, neuronal survival and modulation of synaptic proteins levels (Diniz et al., 2014b). We have previously demonstrated that TGF-β1 induces the formation of synapses between cerebral cortex neurons by regulating the expression, levels and distribution of the synaptic proteins synaptophysin and PSD-95 (Diniz et al., 2012). This mechanism differs from those triggered by the main known astrocytic synaptogenic molecules: thrombospondin, hevin, and glypican-4 (Christopherson et al., 2005; Kucukdereli et al., 2011; Allen et al., 2012).

Here, we determined that the isoforms of TGF-β show heterogeneous expression profiles during the cerebellar development period. In addition, we found that TβRII expression overlap with the synaptic development period of this structure. Corroborating this finding, we showed that TGF-β1 regulates excitatory synapse formation between granular neurons by regulating the expression of synaptic proteins. Together with our previous data, we show that different cues contribute to cerebellar development: whereas EGF modulates CGPs proliferation and neuronal migration (Martinez et al., 2011); TGF-β influences excitatory synapse formation. The identification of factors that govern the cerebellar synaptic circuitry, such as TGF-β, might contribute to the development of therapies for disorders of excitatory and inhibitory input, such as behavioral deficits observed in patients with autism spectrum disorder and deficits in motor control due to ataxia cerebellar.

## Author Contributions

AA: performed experiments; analyzed data, wrote the paper; LD: performed experiments; analyzed data, wrote the paper; CE: performed experiments; analyzed data; BM: performed experiments; analyzed data; RM: performed experiments; analyzed data; FG: analyzed data, wrote the paper.

## Conflict of Interest Statement

The authors declare that the research was conducted in the absence of any commercial or financial relationships that could be construed as a potential conflict of interest.

## Abbreviations

BG: Bergmann glia
BMP: bone morphogenetic protein
BrdU: bromodeoxyuridine
CNS: central nervous system
DAPI: 4′,6-diamidino-2-phenylindole dihydrochloride
DMEM/F12: Dulbecco’s modified Eagle’s medium
EGF: epidermal growth factor
EGL: external granular layer
FBS: fetal bovine serum
FGF: fibroblast growth factor
GCPs: granular cell precursors
GFAP: glial fibrillary acidic protein
IGL: internal granular layer
ML: molecular layer
NCAM: neural cell adhesion molecule
PBS: phosphate-buffered saline
PFA: paraformaldehyde
PCL: Purkinje cell layer
TGF-β1: transforming growth factor beta-1
TGF-β2: transforming growth factor beta-2
TGF-β3: transforming growth factor beta-3
TGF-β: transforming growth factor beta family
TβRI: transforming growth factor receptor type I
TβRII: transforming growth factor receptor type II
