# Supplementary figures and images for: Effects of Transforming Growth Factor Beta 1 in Cerebellar Development: Role in Synapse Formation
Source: Front Cell Neurosci. 2016 Apr 27;10:104. doi: 10.3389/fncel.2016.00104 (PMC4846658; doi:10.3389/fncel.2016.00104)

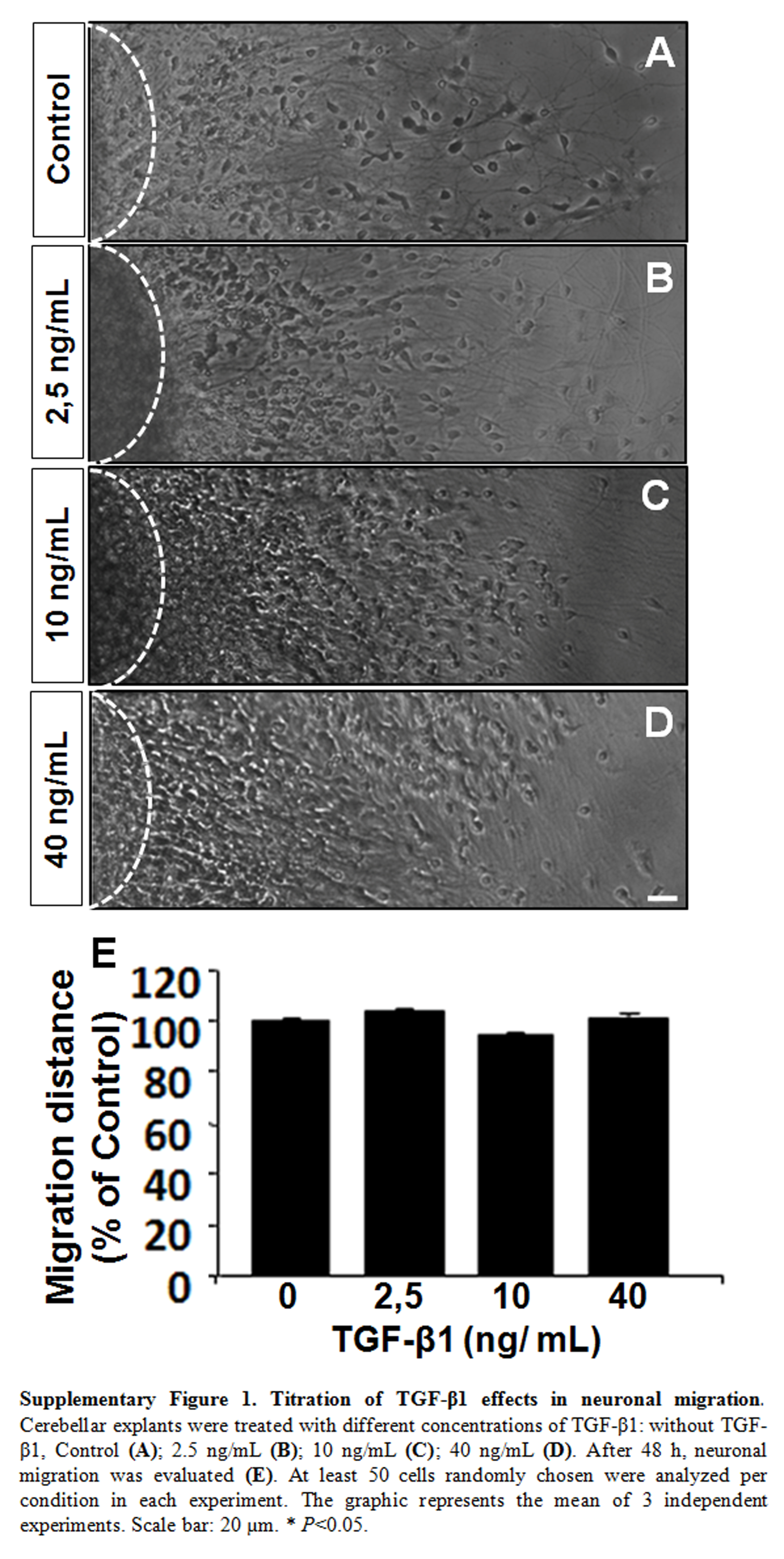

Supplement: Supplementary file 1 [file Image_1.TIF]

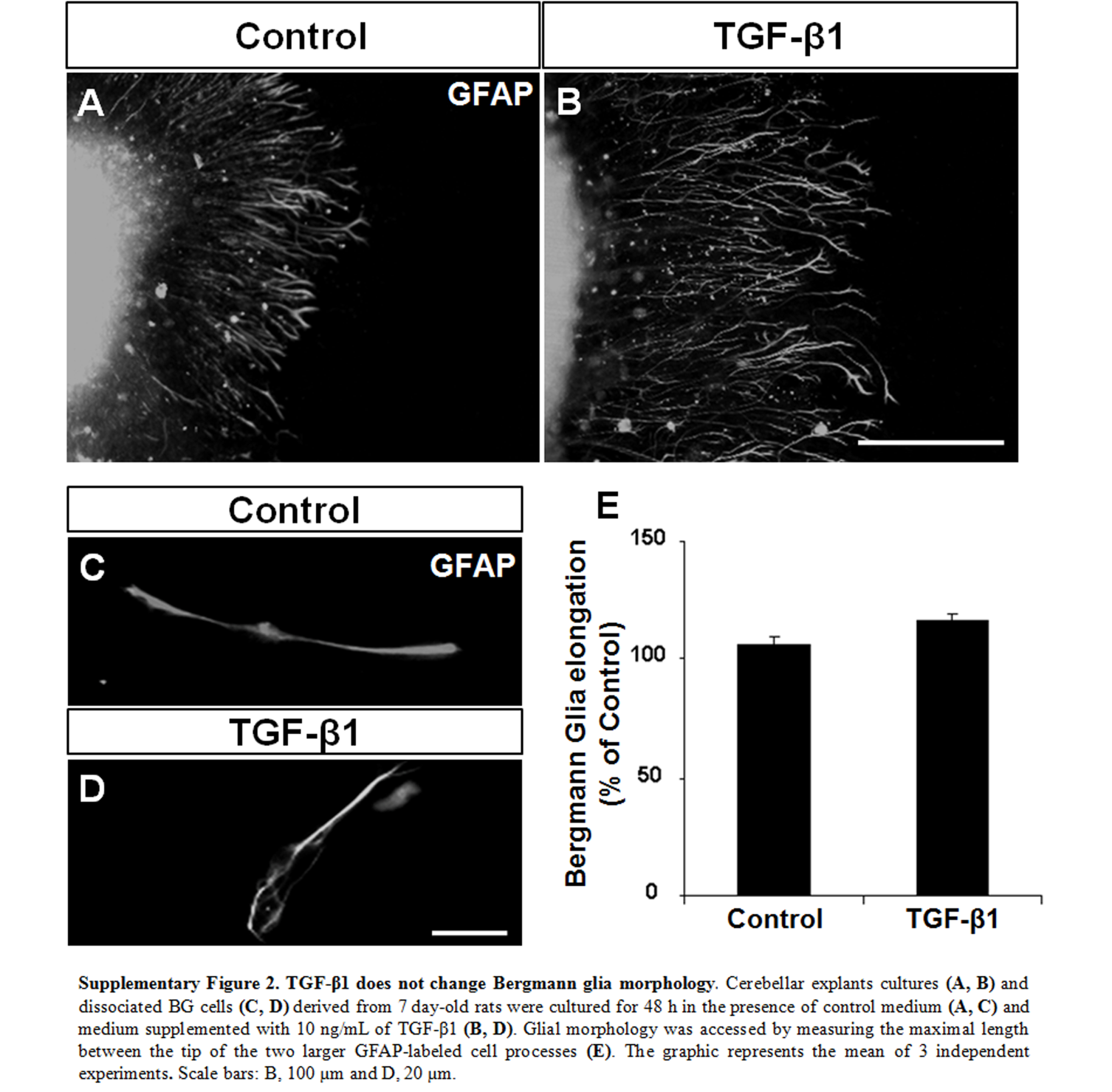

Supplement: Supplementary file 2 [file Image_2.TIF]
